# Primary Metabolism, Phenylpropanoids and Antioxidant Pathways Are Regulated in Potato as a Response to *Potato virus Y* Infection

**DOI:** 10.1371/journal.pone.0146135

**Published:** 2016-01-04

**Authors:** Polona Kogovšek, Maruša Pompe-Novak, Marko Petek, Lena Fragner, Wolfram Weckwerth, Kristina Gruden

**Affiliations:** 1 Department of Biotechnology and Systems Biology, National Institute of Biology, Večna pot 111, Ljubljana, Slovenia; 2 Department of Biology, Biotechnical Faculty, University of Ljubljana, Večna pot 111, Ljubljana, Slovenia; 3 Department of Ecogenomics and Systems Biology, Faculty of Life Sciences, University of Vienna, Althanstrasse 14, Vienna, Austria; 4 Vienna Metabolomics Center (VIME), University of Vienna, Althanstrasse 14, 1090 Vienna, Austria; National Taiwan University, TAIWAN

## Abstract

Potato production is one of the most important agricultural sectors, and it is challenged by various detrimental factors, including virus infections. To control losses in potato production, knowledge about the virus—plant interactions is crucial. Here, we investigated the molecular processes in potato plants as a result of *Potato virus Y* (PVY) infection, the most economically important potato viral pathogen. We performed an integrative study that links changes in the metabolome and gene expression in potato leaves inoculated with the mild PVY^N^ and aggressive PVY^NTN^ isolates, for different times through disease development. At the beginning of infection (1 day post-inoculation), virus-infected plants showed an initial decrease in the concentrations of metabolites connected to sugar and amino-acid metabolism, the TCA cycle, the GABA shunt, ROS scavangers, and phenylpropanoids, relative to the control plants. A pronounced increase in those metabolites was detected at the start of the strong viral multiplication in infected leaves. The alterations in these metabolic pathways were also seen at the gene expression level, as analysed by quantitative PCR. In addition, the systemic response in the metabolome to PVY infection was analysed. Systemic leaves showed a less-pronounced response with fewer metabolites altered, while phenylpropanoid-associated metabolites were strongly accumulated. There was a more rapid onset of accumulation of ROS scavengers in leaves inoculated with PVY^N^ than those inoculated with PVY^NTN^. This appears to be related to the lower damage observed for leaves of potato infected with the milder PVY^N^ strain, and at least partially explains the differences between the phenotypes observed.

## Introduction

In 2012, potato showed the fifth highest production among all of the crops produced worldwide (http://faostat.fao.org/site/291/default.aspx). However, the production of potato is hindered by various environmental and biological factors, with *Potato virus Y* (PVY) being economically the most important potato viral pathogen. PVY belongs to the family *Potyviridae* and was first described over 80 years ago as a causative agent of potato degeneration [1,2]. PVY can cause up to 80% annual losses in potato crop production, and it results in costly certification programmes.

PVY isolates are classified into different groups, where the isolates from the recombinant PVY^NTN^ group are the most devastating; these result in symptoms that include mosaic, chlorotic and necrotic lesions on leaves, and necrotic ringspots on tubers. In contrast, isolates from the PVY^N^ group cause only mild mosaic lesions on potato leaves [3]. The mechanisms behind the potato response to PVY infection that lead to these different symptomatic phenotypes remain to be determined.

As a result of viral infections, plants can show changes to several different metabolic pathways, with photosynthesis as the most frequently studied [4–9]. The metabolism of carbohydrates also alters in response to viral infections, which can be observed as changes in the levels of sugars [10–12] and expression of sugar-metabolism-associated genes [13]. Plants can also respond to viral infection by changes in the production of amino acids, which are needed for successful plant defence responses [6,12,14,15] and for virus multiplication [16]. One of the first responses to infection is activation of reactive oxygen species (ROS), which act as an important regulatory mechanism [17] and which are directly toxic to the pathogens [18]. At the same time, the expression of ROS scavenging mechanisms increases, to reduce the damage to the plant cells caused by the toxic ROS [14,19–21]. When exposed to pathogens, plants produce secondary metabolites [11,22,23] and pathogenesis-related proteins, both of which are involved in plant defences.

The development of symptoms in potato plants inoculated with PVY^NTN^ is still not fully understood, and transcriptome data alone do not allow full insight into plant responses to these infections. The aim of the present study was to analyse the alterations in the metabolome and on gene expression level of asymptomatic PVY^N^-inoculated potato plants in comparison with those of symptomatic PVY^NTN^-inoculated potato plants.

## Results

### Development of symptoms

The mock-inoculated potato leaves of cv. ‘Igor’ did not show any morphological changes. PVY^N^ inoculation triggered no symptoms or very mild symptoms, which were expressed as a few chlorotic or necrotic ring spots at 7 dpi. The PVY^NTN^-inoculated leaves responded to the infection with the development of necrotic lesions on the inoculated leaves between 6 dpi and 7 dpi (S1 Fig). By 13 dpi, most of the inoculated leaves had fallen off, and systemic symptoms in the form of mosaic, necrosis and chlorosis were observed on the upper non-inoculated leaves of PVY^NTN^-inoculated plants. At 26 dpi all lower inoculated leaves from virus inoculated plants had fallen off and all upper non-inoculated leaves were curled. Mock inoculated plants did not show any changes. The successes of virus infection and spread was confirmed on upper leaves by RT-qPCR (data not shown) [24].

Mock-inoculated, PVY^N^-inoculated, and PVY^NTN^-inoculated leaves were harvested at three time points: 1 dpi to observe any early responses to inoculation; 3 dpi to observe responses before symptom development and 6 dpi when the symptoms were developed. At 3 dpi and 6 dpi, upper non-inoculated leaves were also harvested. These upper leaves from 1 dpi were not included in the study as previous data have shown that the virus does not spread to upper leaves before 3 dpi [25].

### Overview of metabolic changes in the inoculated leaves of potato plants after infection with PVY

The polar metabolites from the inoculated potato leaves were analysed by GC-MS. In all, 168 peaks were apparent in the chromatograms for the inoculated and non-inoculated leaves (S1 Table, S2 Fig). Seventy of these compounds were annotated using spectral match factors over 850 and comparisons of retention indices, while others are indicated as unknown and specified according to their retention times, retention indices and specific *m/z* values. The metabolites identified were linked with their metabolic pathway ontologies for easier interpretation (S2 Table).

**Fig 1:**
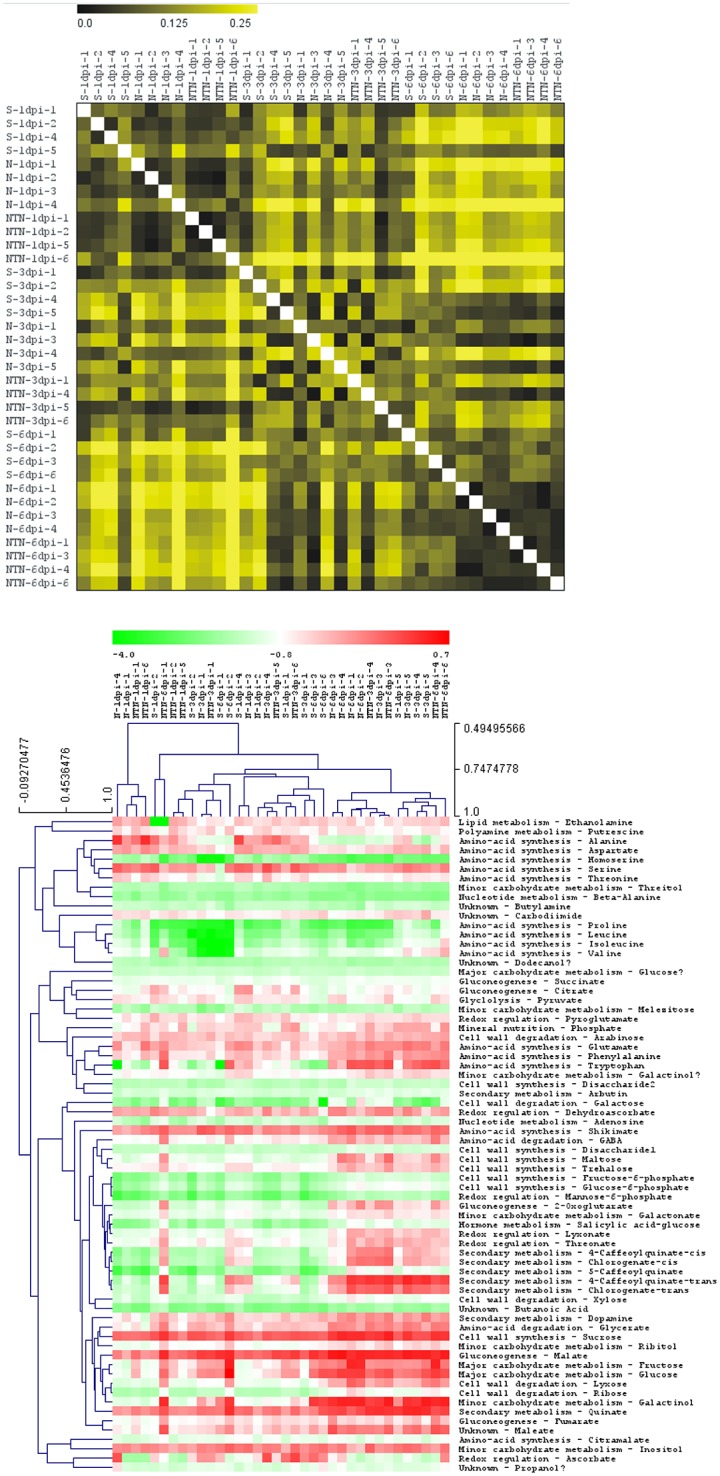
Overview of the response of individual leaf sample and changes of metabolites in inoculated leaves. Mock-inoculated (S, 1 to 6), PVY^N^-inoculated (N, 1 to 6), and PVY^NTN^-inoculated (NTN, 1 to 6) leaves, collected at 1, 3 and 6 dpi, are shown. Distance matrix (A) shows more similar responses between samples collected at 1 dpi and at 6 dpi, while less uniform response is observed in samples collected at 3 dpi. Hierarchical clustering (B) was done on a set of identified metabolites to which metabolite ontology (metabolic pathway) was assigned. Clusters of metabolites linked to amino-acid synthesis and those linked to secondary metabolism and cell wall clustered together.

For an overview of the data structure, the distance matrix calculations were done on the complete dataset (Fig 1A). The distance matrix shows high variability in the responses at 3 dpi, while more uniform responses were seen at 1 dpi and 6 dpi. At 1 dpi, regardless of the treatments, all of the plants had very similar responses and at 6 dpi, the virus-inoculated leaves had different responses from the mock-inoculated leaves. Further, hierarchical clustering was applied to the set of seventy identified metabolites to observe and compare accumulation pattern of individual metabolite in each sample (Fig 1B). Clusters of metabolites linked to amino-acid synthesis are clearly separated from the clusters predominated with metabolites linked to secondary metabolism and cell-wall synthesis/degradation.

The different time-courses of the disease responses of the individual potato plants collected at 1 dpi and 6 dpi were further shown by PCA (S3 Fig). The first two principal components explained 58% of the overall variance. The analysis showed time-dependent separation of the virus inoculated samples (first component, 47% of the variance). Mock-inoculated samples were separated from the virus inoculated samples and between both time points by the second component.

Two-way ANOVA analysis was used to identify the metabolites with statistically significant changes (p<0.01) in relation to time and treatment. Here, 83 metabolites (32 of known identity) showed significant changes in relation to time (S2 Table). Also, three metabolites showed significant changes relative to the treatments: aldopentose, and two unidentified metabolites. Finally, 31 metabolites showed significant changes for the interaction between time and treatment, among which there were GABA, α-ketoglutarate, glycerate, maleate, maltose, phenylalanine, pyruvate, succinate, sucrose, and valine (S2 Table).

**Fig 2:**
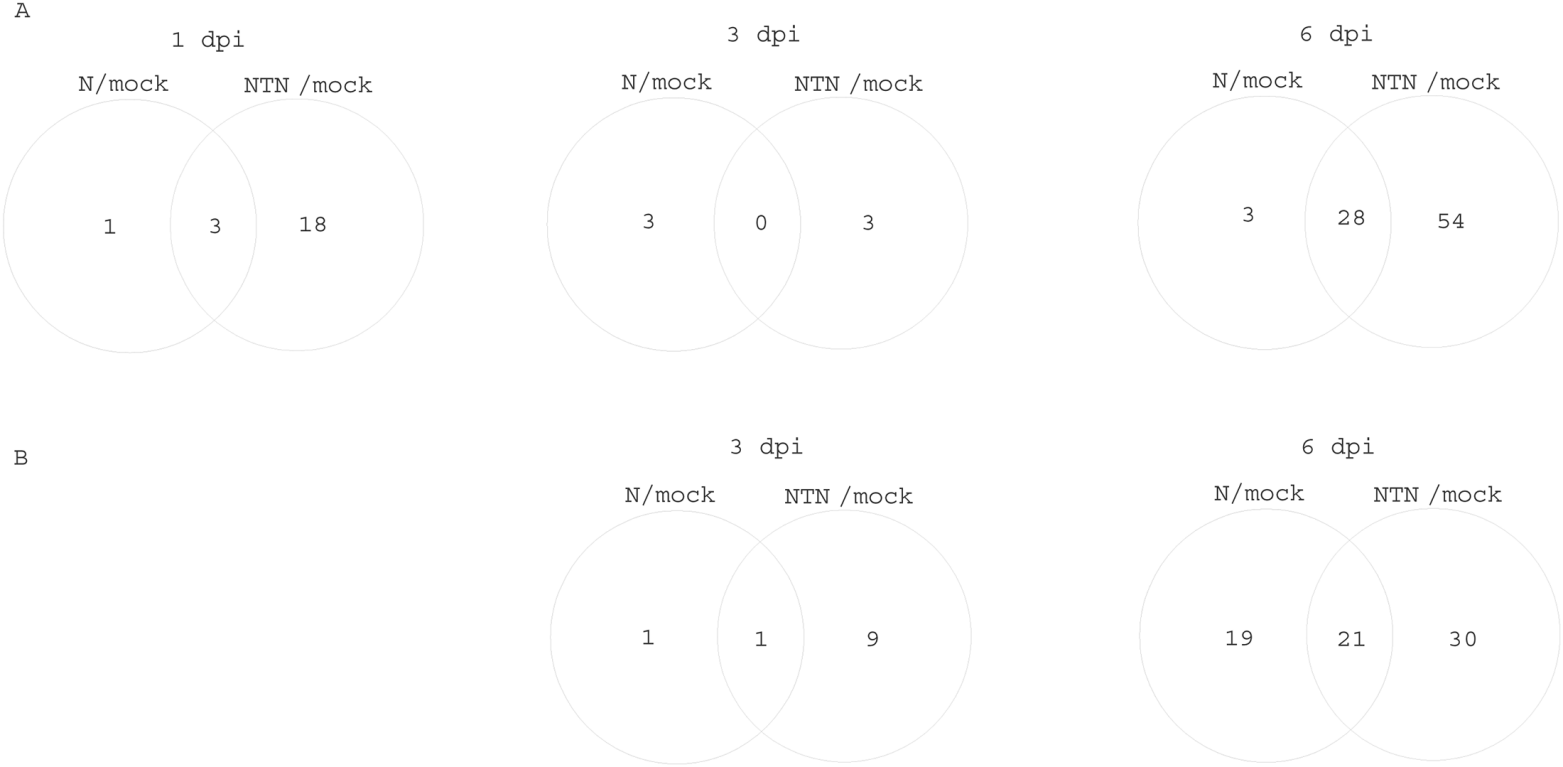
Venn diagrams showing the numbers of statistically significantly differentially accumulated metabolites at the different times in the inoculated leaves (A) and non-inoculated leaves (B). Diagrams are showing the number of metabolites in PVY^N^-inoculated and PVY^NTN^-inoculated leaves, relative to the mock-inoculated leaves (N/mock, NTN/mock, respectively). The intercept areas show the numbers of metabolites that were common to both of the plant responses in the comparisons.

The analysis with t-tests showed that in the inoculated leaves, a total of 100 peaks changed significantly for at least one of the times or treatments; however, only 41 of these peaks were identified as known metabolites (S2 Table). The highest number of metabolites that showed significant changes was seen between the mock-inoculated leaves and the virus-inoculated leaves at 6 dpi, followed by 1 dpi and 3 dpi, where the highest number of metabolites showed changes in the PVYNTN-inoculated leaves, followed by the PVYN-inoculated leaves, relative to the mock-inoculated leaves (Fig 2A, S2 Table).

When comparing the levels of the metabolites in the leaves inoculated with one of the virus isolates relative to the mock-inoculated leaves, the metabolite concentrations were generally lower in the virus-inoculated leaves at 1 dpi (S2 Table). For 11% of all of the metabolites detected, the changes in their levels were statistically significant. In contrast, at 6 dpi, concentration of 17% and 48% of the metabolites was significantly higher in the PVY^N^-inoculated leaves and in the PVY^NTN^-inoculated leaves, respectively, relative to mock inoculated leaves.

### Analysis of changes in the metabolite concentrations in relation to the metabolic pathways

Metabolites involved in primary metabolism processes were examined into more detail, in particular for major carbohydrate metabolism, amino-acid metabolism, and the TCA cycle. The metabolites associated with secondary metabolism also showed interesting patterns of accumulation over the observed times, such as for the phenylpropanoids and the redox-state-related metabolites.

The concentrations of glycerate, sucrose, succinate and threonate showed statistically significant decreases at the beginning of disease development (1 dpi) in the PVY^NTN^-inoculated leaves, relative to the mock-inoculated leaves (Table 1). The sucrose concentration showed a significant decrease also in response to the inoculation with PVY^N^. Similar patterns of decreases were generally observed also for the majority of the metabolites in the virus-inoculated leaves at 1 dpi. At 3 dpi, the concentrations of citrate and succinate showed statistically significant increases in the leaves inoculated with PVY^N^ and PVY^NTN^, respectively, relative to the mock inoculations. Valine and the metabolites linked to the phenylpropanoid pathway were the most greatly accumulated at 6 dpi in both of the PVY-inoculated leaves, relative to the mock-inoculated leaves.

**Table 1:** Changes in the metabolite concentrations following mock inoculation and PVY inoculations.

Metabolic pathway	Metabolite	Time post infection (dpi)					
		1		3		6	
		N:mock	NTN:mock	N:mock	NTN:mock	N:mock	NTN:mock
**Gaba shunt**	GABA	-0.51	-0.57	-0.11	0.09	1.11*	1.47*
	Glycerate	-0.99	-1.13*	0.31	0.02	0.87*	1.22**
**Amino-acid synthesis**	Citramalate	-0.20	-0.70	0.25	0.52	0.29	0.66*
	Glutamate	-0.20	-0.13	0.16	-0.22	0.64	1.16*
	Phenylalanine	-0.17	-0.33	0.19	0.02	1.28**	2.11***
	Shikimate	-0.50	-0.33	0.61	0.47	0.42	0.55*
	Tryptophan	-0.02	-1.67	-0.25	-0.66	1.64	2.23*
	Valine	-0.05	-0.46	-0.08	-0.99	2.26**	4.42*
	Threonine	0.33	-0.10	-0.02	-0.42	0.31	0.85*
**Major carbohydrate**	Fructose-6-P	-1.60	-1.78	-0.03	-0.69	1.83	1.68*
	Glucose-6-P	-1.86	-1.97	-0.09	-0.81	1.81	1.66*
	Maltose	-1.13	-0.83	0.33	-0.88	2.89*	3.18*
	Sucrose	-0.43*	-0.45*	0.31	0.00	0.82*	0.83*
**TCA cycle**	2-Oxoglutarate	-1.09	-1.66	1.18	0.05	3.44*	3.70**
	Citrate	-0.48	-1.15	0.68*	1.23	0.18	1.34
	Succinate	-0.52	-0.52*	0.20	0.31*	0.88*	1.04***
	Pyruvate	-0.01	-0.53	0.54	0.54	1.27	1.76**
**ROS scavengers**	Dehydroascorbate	-0.36	-0.27	0.67	-0.38	0.94	0.98*
	Lyxonate	-0.82	-0.99	0.18	-0.32	1.59	1.78**
	Mannose-6-P	-1.86	-1.90	-0.06	-0.81	1.78	1.55*
	Pyroglutamate	-0.42	-0.57	0.02	-0.07	0.24	0.41*
	Threonate	-0.96	-1.22*	0.31	0.00	1.38	1.86*
**Phenylpropanoid**	4-Caffeoylquinate-*trans*	-1.98	-4.44	0.15	-0.67	2.39	2.83**
	Chlorogenate-*trans*	-1.56	-3.00	0.37	-0.53	2.58	2.91**

The responses in the leaves to inoculation with PVY^N^ and PVY^NTN^ are compared to the mock inoculation (N:mock, NTN:mock, respectively). The log2 ratios of the metabolite concentrations at the different times (i.e., 1, 3, 6 dpi) are shown. The corresponding metabolic pathways were assigned. The statistical significances of the changes in metabolite levels were evaluated (*t*-tests).

*, *p* <0.05;

**, *p* <0.01;

***, *p* <0.001.

Several unidentified metabolites also showed interesting patterns of accumulation. Indeed, these might be important in the potato responses to PVY infection; however, chemical identification would be needed to provide a full biological interpretation of their roles (S2 Table).

### Integration of the metabolome data with response at the gene expression level

**Fig 3:**
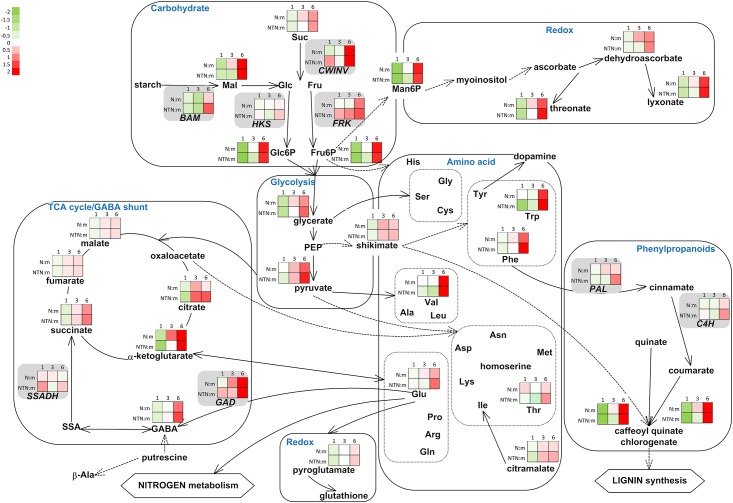
Integration of the changes in the metabolites and transcripts associated with selected metabolic pathways in the PVY^N^-inoculated and PVY^NTN^-inoculated leaves, relative to the mock-inoculated leaves. The metabolites identified and the genes analysed from the primary and secondary metabolism and from redox reactions are shown. Each coloured square represents the log2 ratios of the expression or abundance (red, high; green, low) at 1, 3 and 6 dpi in the PVY^N^-inoculated (N:m) and PVY^NTN^-inoculated (NTN:m) leaves, relative to the mock-inoculated leaves (as indicated). MapMan BINs linked to primary metabolism: 2.1.1 major CHO metabolism, sucrose synthesis; 2.2.1 major CHO metabolism, sucrose degradation; 2.2.2 major CHO metabolism, starch degradation. MapMan BINs linked to the GABA shunt: 13.1.1.1 amino-acid metabolism, GABA synthesis; 8.1. TCA; 22. polyamine metabolism; 12.2.1002 N-metabolism, ammonia. MapMan BINs linked to secondary metabolism: 16.2.1 secondary metabolism, phenylpropanoids biosynthesis; 13.1. amino-acid synthesis.

Gene expression analysis of the selected genes involved in the metabolic pathways indicated in Table 1 was used to provide a better understanding of the potato response to PVY infection in inoculated leaves. Integration of the gene expression and metabolome data using MapMan ontology showed changes in major carbohydrates metabolism, the GABA shunt, phenypropanoid metabolism, and antioxidant metabolism (Fig 3).

Expression of cell-wall invertase (*CWINV*) decreased in the PVY^N^-inoculated leaves, relative to the mock-inoculated leaves at 1 dpi (Fig 3, S3 Table). At the same time, expression of fructokinase (*FRK*) increased in the PVY^NTN^-inoculated leaves. The PVY^NTN^-inoculated leaves also showed an increase in the expression of *CWINV*, hexokinase (*HKS*) and β-amylase (*BAM3*) at 6 dpi, relative to the mock-inoculated leaves.

Expression of the glutamate decarboxylase (*GAD*) gene that codes for one of the three most important GABA-shunt enzymes increased through disease development in the leaves inoculated with both of the PVY isolates, in comparison to the mock-inoculated leaves (Fig 3, S3 Table), while the gene for succinic semialdehyde dehydrogenase (*SSADH*) had a unique pattern of expression through disease development.

Genes for phenylalanine-ammonia lyase (*PAL*) and cinnamate 4-hydroxylase (*C4H*) had similar expression patterns through disease development (Fig 3, S3 Table).

### Responses in the upper non-inoculated leaves

To broaden our understanding of the potato response to these PVY infections, the metabolites in upper non-inoculated leaves were also analysed at 3 dpi and 6 dpi (S1 Table). Two-way ANOVA showed that in the upper non-inoculated leaves, 51 metabolites (21 identified) showed significant changes in relation to time, and 10 metabolites (5 annotated: carbodiimide, melezitose, shikimate, fructose 6-phosphate, β-alanine) in relation to treatment (S2 Table). Four metabolites (two identified: dehydroascorbate, β-alanine) showed significant changes due to both of these factors (i.e., time and treatment).

Complementary analysis with t-tests showed that the responses were less intensive in these non-inoculated leaves compared to the PVY-inoculated leaves. In all, 84 metabolites (33 identified) showed statistically significant changes for at least one of the times or treatments (S2 Table). As was seen for the PVY-inoculated leaves, at 6 dpi, the non-inoculated leaves also had higher numbers of metabolites that showed changes in the PVYNTN-inoculated plants over the PVYN-inoculated plants, relative to the mock-inoculated plants (Fig 2B).

As for the PVY-inoculated leaves, selected metabolic pathways were examined in more detail also in these upper non-inoculated leaves. At 3 dpi, the shikimate concentrations in these upper non-infected leaves showed statistically significant higher levels in both the PVY-inoculated plants, and dehydroascorbate showed significant accumulation in the non-inoculated leaves of the PVY^NTN^-inoculated plants (Table 2). Later, at 6 dpi, almost all of the metabolites related to amino-acid metabolism and the phenylpropanoid pathway accumulated in these upper leaves of the plants inoculated with both virus isolates, which was most prominent for the phenylpropanoids. In contrast, the concentrations of citramalate, malate and *myo*-inositol showed statistically significant decreases in these upper leaves of the plants inoculated with both of the virus isolates, relative to the mock-inoculated plants.

**Table 2:** Changes in the metabolite concentrations in the upper non-inoculated leaves following mock inoculation and PVY inoculation.

Metabolic pathway	Metabolite	Time post infection (dpi)			
		3		6	
		N:mock	NTN:mock	N:mock	NTN:mock
**Amino-acid synthesis**	Valine	-0.12	-0.44	0.72*	1.26*
	Isoleucine	-0.45	-1.66	2.12	2.52*
	Shikimate	0.37**	0.37*	0.09	0.36
	Citramalate	0.23	0.06	-0.39	-0.45*
**TCA cycle**	2-Oxoglutarate	0.02	-0.46	2.24**	2.18**
	Malate	0.09	0.03	-0.34	-0.65*
	Citrate	0.4	0.5	0.4	0.8*
**ROS scavengers**	Lyxonate	-0.12	-0.12	1.37**	1.41**
	Dehydroascorbate	0.61	0.83*	-0.36	-0.67
	Inositol	0.2	0.25	-0.68*	-0.35
**Phenylpropanoid**	Chlorogenate-*cis*	0.26	-0.06	2.64**	2.76**
	5-Caffeoylquinate	0.19	-0.72	3.26	3.23*
	Chlorogenate-*trans*	0.39	0.19	2.01**	2.1**
	4-Caffeoylquinate-*cis*	0.25	-0.12	2.7**	2.81**

The responses in the leaves to inoculation with PVY^N^ and PVY^NTN^ are compared to the mock inoculation (N:mock, NTN:mock, respectively). The log2 ratios of the metabolite concentrations at the different times (i.e., 3, 6 dpi) are shown. The corresponding metabolic pathways were assigned. The statistical significances of the changes in metabolite levels were evaluated (*t*-tests).

*, *p* <0.05;

**, *p* <0.01.

**Fig 4:**
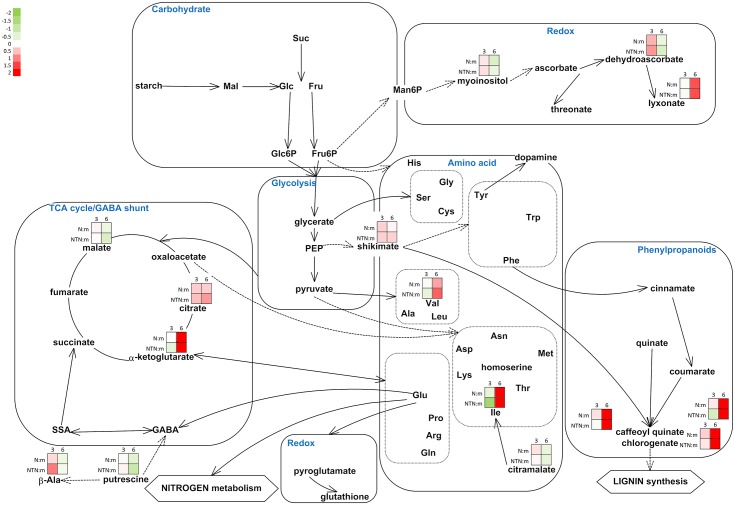
Integration of the changes in the metabolites associated with selected metabolic pathways in the upper non-inoculated leaves from the PVY^N^-inoculated and PVY^NTN^-inoculated plants, relative to the mock-inoculated plants. The metabolites identified from the primary and secondary metabolism and from redox reactions are shown. Each coloured square represents the log2 ratios of the concentration (red, high; green, low) at 3 and 6 dpi in the upper non-inoculated leaves of the PVY^N^-inoculated (N:m) and PVY^NTM^-inoculated (NTN:m) plants, relative to the mock-inoculated plants (as indicated). MapMan BINs linked to primary metabolism: 2.1.1 major CHO metabolism, sucrose synthesis; 2.2.1 major CHO metabolism, sucrose degradation; 2.2.2 major CHO metabolism, starch degradation. MapMan BINs linked to the GABA shunt: 13.1.1.1 amino-acid metabolism, GABA synthesis; 8.1. TCA; 22. polyamine metabolism; 12.2.1002 N-metabolism, ammonia. MapMan BINs linked to secondary metabolism: 16.2.1 secondary metabolism, phenylpropanoids biosynthesis; 13.1. amino-acid synthesis.

The data for the metabolome study in these upper non-inoculated leaves were also defined according to the MapMan ontology, and visualised (Fig 4).

## Discussion

Responses of potato plants to infection with PVY have until now been studied at different levels, from morphological to biochemical, and from proteomic to transcriptomic [3,9,26,27]. Even though early alterations in potato metabolism that precede symptom development and virus multiplication have been monitored, the processes involved in symptom development have not yet been well explained. In our previous study of PVY^N^-inoculated and PVY^NTN^-inoculated leaves of the sensitive potato cultivar ‘Igor’, potato microarrays were used to identify genes involved in early responses (0.5 hpi, 12 hpi, 2 dpi) to inoculation [13]. In the present study, similar experiments were performed to observe changes at the transcriptional and metabolome levels at 1, 3 and 6 dpi and correlate them with symptoms development. Strong distinctive symptoms appeared on the PVY^NTN^-inoculated leaves, while on PVY^N^-inoculated plants, only mild or no symptoms were observed at 7 dpi.

Three different approaches were employed to get a general overview of the response of individual potato leaves and changes in metabolic pathways after inoculation with PVY isolates. The distance matrix and hierarchical clustering show high variability between plants in the response at 3 dpi, which is most probably the consequence of different disease response rates in the individual potato plants (Fig 1). At 1 and 6 dpi, responses of virus-inoculated plants can be distinguished from mock-inoculated plants, and even more PCA plot shows separation of samples inoculated with different PVY isolates at 6 dpi (S3 Fig). Interestingly, mock-inoculated samples separate from the virus inoculated samples at 1 and 6 dpi, reflecting the changes in developmental stage of the plants [28].

### Changes in sugar balance in response to virus infection

Soluble carbohydrates and starch have been shown to have important roles in plant responses to virus infections, where different biological systems have their specific modes of response (e.g., for different types of plant—virus interactions). In PVY-inoculated potato leaves, a two-stage response was observed for the sugar concentrations, with an initial decrease followed by a strong increase. Decreased concentrations of sugars at 1 dpi might be the consequence of soluble sugar uptake for starch synthesis (Fig 3) which is supported by increased expression of the *GBSS* gene in the PVY^NTN^-inoculated leaves, relative to the mock-inoculated leaves [13]. In tomato infected with *Tomato mosaic virus* (ToMV; Tobamovirus), the concentrations of sucrose and glucose were in opposite proportions, with glucose being in abundance in the inoculated asymptomatic leaves [11]. Similar relationships between sucrose and glucose were detected also in both the symptomatic and asymptomatic PVY-inoculated potato leaves at 6 dpi. Alterations in sugar abundance, together with metabolic feedback inhibition of photosynthesis in source leaves, indicate a source-to-sink transition that further affects the mechanism of sugar transport and partitioning at the whole plant level [29]. Accumulation of sugars in PVY-inoculated potato leaves could be a consequence of the source-to-sink transition of those leaves. The idea is additionally supported by increase in the expression of genes for sink-specific enzymes, such as *GBSS* [13] and *CWINV* in PVY-inoculated leaves. However, no direct functional confirmation is available to support this hypothesis.

There were no clear differences at 6 dpi in the levels of the soluble sugars in the asymptomatic PVY^N^-inoculated and symptomatic PVY^NTN^-inoculated leaves. However, differences in the dynamics of the responses between the PVY^N^-inoculated and PVY^NTN^-inoculated potato leaves were detected at the gene expression level in this study and previous [13] study. Our results indicate that the dynamics of the responses in terms of the rates of activation of transcription and metabolite accumulation have important roles in symptom development. Indeed, significantly more soluble sugars accumulated in the symptomatic PVY^NTN^-inoculated leaves than in the asymptomatic PVY^N^-inoculated leaves (see also cell wall synthesis and degradation, S2 Table), which shows the importance of these sugar-associated responses of the leaves under infection with the more aggressive virus isolate (i.e., PVY^NTN^).

In these studied potato—PVY interactions, no changes were seen, however, for the sugar balance in the upper non-inoculated leaves over the time period of this study (Fig 4). Albeit, in the later stages of disease development altered sugar concentrations can be expected in these upper non-inoculated leaves, which has been partially indicated by the changed expression of *GBSSI* in upper leaves of the PVY-infected potato cv. ‘Igor’ [25]. Soluble sugars were generally reported to accumulate in non-inoculated leaves of infected plants, while levels of starch changed in different direction upon infection [5,10,14,16]. Interestingly, higher concentrations of carbohydrates were detected in the asymptomatic leaves compared to the symptomatic leaves [10].

### Alterations in the Krebs cycle and GABA-shunt activities due to virus infection

An often-observed characteristic of plant responses to virus infections is a decrease in photosynthesis and its associated mechanisms [5,7,8,26,27,30]. Due to the lower energy production, the respiration rate increases to provide resources for the defence responses (and/or for pathogen multiplication). The induction of genes related to cellular respiration has been shown in compatible interactions of *Arabidopsis* plants with various viruses at the late stages of infection [30] and accumulation of Krebs cycle intermediates was observed also in a study of the susceptible interactions between ToMV and tomato [11]. In this study Krebs cycle intermediates accumulated to higher levels in PVY-inoculated potato leaves at 6 dpi relative to mock-inoculated leaves (Fig 3). Similar responses, albeit less pronounced, were also observed in the upper non-inoculated potato leaves. TCA intermediates accumulated in the upper leaves of tobacco infected with TMV [16] and in *Amaranthus hypochondriacus* L. infected with *Ageratum enation virus* (AEV) [12].

The Krebs cycle is directly affected by oxidative stress, and plants have the possibility to avoid the Krebs cycle via the GABA shunt, which uses less sensitive enzymes and to maintain the required NADH levels [31]. In transgenic rice seeds and tomato plants, direct connections between the expressions of genes for GABA synthesis (i.e., *GAD*) and GABA catabolism and the levels of GABA have been shown [32,33]. The data from the present study support this model, as the patterns of expression of *GAD* was in agreement with the GABA precursor (glutamate) and GABA concentrations. Expression of the *SSADH* transcript was shown not to be significantly affected by the inoculation with these PVY isolates in potato plants. The *SSADH* products are succinate and NADH, and these are substrates for the mitochondrial respiratory chain, and they have roles in the prevention of accumulation of ROS, and the consequent cell death. Therefore, other levels of regulation are expected here [34]. One of the potential mechanisms of regulation of transcription is via micro (mi)RNAs, which has been demonstrated in TMV-infected tobacco plants, where a decrease in miR408 expression resulted in an increase in the concentrations of the Krebs cycle intermediates [16].

GABA is involved in different processes that are connected to abiotic and biotic stress, presumably through different pathways [12,35–37]. The GABA-shunt metabolites and genes have been shown to have important roles not only in the Krebs cycle, but also for the central carbon/ nitrogen metabolism [32,38,39]. This occurs mainly via glutamate, which has crucial roles in amino-acid metabolism, assimilation of nitrogen, ROS scavenging, and other metabolic functions important for plant responses to infection [40]. In infected plants, stronger demands for carbon can shift amino acids into energy-generating pathways. In TMV-infected upper leaves of tobacco plants, the concentrations of amino acids were reported to decrease, and this decrease paralleled virus accumulation [16]. In ToMV-infected lower and upper tomato leaves, and in TRV-infected upper *Arabidopsis* leaves, some amino acids were reported to decrease and some to increase [11,41]. In the present study, we observed clustering of metabolites linked to amino-acid synthesis showing co-accumulation of several amino-acid (Fig 1B) which stresses the importance of the amino-acid metabolism in potato response to infection [31,42]. The initial decrease in amino acid concentrations in the potato leaves inoculated with both of PVY isolates was followed by strong accumulation of amino acids and their precursors (Fig 3). Whether the amino acids accumulation pattern in PVY-inoculated potato has a role in plant defence response [31,42] or in promotion of disease development [43], needs to be elucidated.

Glutamate also has an important role in ROS scavenging mechanisms, via glutathione and the production of NO and polyamines [40], e.g., putrescine, which significantly decreased at 6 dpi in the upper non-inoculated potato leaves in the present study (Fig 4). Putrescine is further used in GABA and β-alanine synthesis, and accumulation of β-alanine was seen at 3 dpi in the upper non-inoculated leaves, which has been shown to be required for tolerance to abiotic stress [44]. Similar pattern of putrescine and β-alanine accumulation were reported previously in non-inoculated leaves of TMV-infected tobacco plants [16], and in TRV-infected *Arabidopsis* plants [41].

### Potato accumulates ROS scavenging metabolites in response to PVY inoculation

One of the first and most important protection and signalling mechanisms that is induced in infected plants is the production of ROS [19,20], and consequently the accumulation of ROS-scavenging enzymes and metabolites [45]. In the case of incompatible interactions, ROS presumably act against pathogens, and lead to the formation of necrosis, in a process known as the hypersensitive reaction [46]. However, ROS-associated metabolites and genes have also been observed to increase in infected and systemic leaves in compatible interactions. In PVY-inoculated potato leaves, the levels of glutathione and ascorbate were not significantly changed, while their precursors and degradation products showed statistically significant changes in PVY-inoculated potato leaves, compared to the mock-inoculated leaves. In general all of the metabolites linked to antioxidant metabolism initially decreased, while they increased through the disease development in the potato leaves inoculated with both of the PVY isolates (Fig 3). More intensive accumulation of ROS-associated proteins was detected when comparing asymptomatic and symptomatic tomato plants infected with TYLCV [14]. In the present study, at 6 dpi, glutathione precursor glutamate accumulated to higher levels in PVY^NTN^-inoculated potato leaves than in PVY^N^-inoculated ones. Moreover, at 3 dpi, there was greater accumulation of dehydroascorbate in the PVY^N^-inoculated leaves than in the PVY^NTN^-inoculated leaves. On the other hand, a strong early increase in the expression of the genes related to antioxidant metabolism was previously described in the PVY^NTN^-inoculated potato leaves of cultivars Igor [13] and Premier Russet [9]. Interestingly, no expression of those genes was detected in response of resistant cultivar Premier Russet to PVY^O^ inoculation [9].

In the upper non-inoculated potato leaves, the responses were not so uniform, with only dehydroascorbate showing accumulation at 3 dpi, and lyxonate at 6 dpi. Similarly, the accumulation of intermediates in glutathione and ascorbate metabolism was observed in systemic leaves of SuCMoV-infected sunflowers [21], and decreases or no changes in the concentrations of certain redox-associated metabolites were observed in non-inoculated leaves of TMV-infected tobacco plants [16].

### Phenylpropanoids accumulate in response to virus infection

Synthesis of phenylpropanoids starts with the shikimate pathway, which has been shown to be responsive to biotic stress [47]. The final products of the shikimate pathway are the aromatic amino acids, which have essential roles in plant metabolism and serve as precursors for a variety of hormones and aromatic secondary metabolites.

Shikimate and the aromatic amino acids phenylalanine and tryptophan accumulated in PVY-inoculated potato leaves at 6 dpi (Fig 3). Changes in the concentrations of phenylalanine and tryptophan were previously detected in tomato plants inoculated with ToMV [11] and TYLCV [14], and accumulation of phenylpropanoids [28,48–50] and associated transcripts [9] has been described for various plant—pathogen interactions. In PVY-inoculated potato leaves, 3-*trans*-caffeoylquinic acid and 4-*trans*-caffeoylquinic acid showed significant accumulation (Fig 3).

In the upper non-inoculated potato leaves, shikimate, 3-*trans*-, 4-*trans*- and 5-*trans*-caffeoylquinic acid accumulated as well (Fig 4). Accumulation of phenylpropanoids and transcripts involved in secondary metabolism has previously been shown in systemically infected symptomatic cucumber and melon plants [50], and in systemically infected tomato leaves [11]. On the other hand, in *Pepino mosaic virus* (PepMV; Potexvirus)-infected tomato, expression of the genes associated with the phenylpropanoid pathway decreased [22]. Phenylpropanoids have been shown to contribute to plant resistance to pathogen infection [23,51], and a gradual increase in 5-caffeoylquinic acid levels was observed in resistant tobacco infected with TMV [48]. In the present study, a faster onset of phenylpropanoids accumulation was observed in the PVY^N^-inoculated leaves, which might be at least partially accountable for the milder symptoms in these leaves, compared to those with PVY^NTN^ inoculation. Similarly, differences in early expression (up to 10 hpi) of phenylpropanoid related transcripts were shown in potato cv. ‘Premier Russet’ response to two PVY isolates, PVY^NTN^ and PVY^O^ [9].

The process of disease development was studied at the gene expression and metabolome levels in the potato—PVY interaction. In PVY-infected potato leaves, the levels of carbohydrates and ROS scavengers initially decreased (at 1 dpi) and then later increased (at 6 dpi), and the responses were similar in both the asymptomatic PVY^N^-infected and symptomatic PVY^NTN^-infected leaves. Similar dynamics were observed also for the abundance of several amino acids, for the GABA shunt and TCA cycle intermediates, and for the phenylpropanoids. The accumulation of these metabolites coincided with the expected onset of virus multiplication. The upper non-inoculated leaves were not yet fully infected at 6 dpi. As expected, the differences in the metabolite profiles in these upper non-inoculated leaves were less pronounced compared to the differences in the PVY-inoculated leaves. However, by 6 dpi, the phenylpropanoids and the amino acids valine and isoleucine accumulated to high levels also in these upper non-inoculated leaves. The comparisons between the responses to each of these PVY isolates in inoculated leaves reveal a more rapid onset of accumulation of ROS scavengers in the leaves inoculated with PVY^N^ than in those inoculated with PVY^NTN^ (i.e., already seen at 3 dpi). This could be connected with the lower damage seen for the leaves of the potato plants infected with this milder PVY^N^ strain, and at least partially explain the differences in the phenotypes observed.

## Experimental Procedures

### Plant material, inoculation and sampling

Potato plants (*Solanum tuberosum*) cv. ‘Igor’ were grown from node tissue cultures and planted in soil in growth chambers that were kept at 21 ±2°C, with a photoperiod of 16 h and relative humidity of 70%. After 4 weeks, three basal leaves were mechanically inoculated with the sap of healthy plants (mock inoculated), PVY^N^-infected plants (N-RB isolate; AJ390285), or PVY^NTN^-infected plants (NIBNTN isolate; AJ585342). The inoculums were left on leaves for 10 min and afterwards washed off with tap water. At 1, 3 and 6 dpi, the inoculated leaves from four plants per time point per treatment were harvested. At 3 and 6 dpi, three upper non-inoculated leaves were also harvested from the same plants. The harvested leaves were stored in tubes and immediately frozen in liquid nitrogen. This plant material was stored at -80°C prior to further analysis. A separate group of six inoculated plants was left for symptom observation.

### Total RNA isolation

Total RNA was isolated from the third inoculated leaf (counting from the ground), using up to 300 mg frozen homogenised leaf material. MagMAX-96 Total RNA Isolation kits (Ambion) and KingFisher apparatus (Thermo) were used, following the manufacturer protocols. The isolated RNA was treated with DNase, quantified with NanoDrop spectrophotometer, and checked for quality by gel electrophoresis, as previously described [13].

### Reverse transcription—quantitative real-time PCR

Up to 2 μg total RNA was transcribed into cDNA using High-Capacity cDNA Reverse Transcription kits (ABI), following the manufacturer recommendations. The final volume of the reaction mixture was 25 μl, and random hexamer primers were used.

RT-qPCR was used to analyse the PVY RNA [24] and expression of the transcripts encoding β-amylase (*BAM*), fructokinase (*FRK*), hexokinase (*HKS*), glutamate decarboxylase (*GAD*), succinic semialdehyde dehydrogenase (*SSADH*), phenylalanine-ammonia lyase (*PAL*), cinnamate 4-hydroxylase (*C4H*), and cell-wall invertase (*CWINV*; [52]) (Table 3). More details on RT-qPCR assays are available in S4 Table.

**Table 3:** Target sequences, forward (FP) and reverse (RP) primers, and probe sequences (Probe) of amplicons for the genes investigated.

Gene	Target sequence ID	Primer sequence (5’– 3’)	Amplicon length(nt)	Amplification efficiency(%)	Source
**BAM**	AF393847^a^; Sotub08g023010 ^b^	FP: TGTAAAGATGTGTCAAGAACATGGATT; RP: AATCTCCAACATTTCCTCCACACT; Probe: AGCTTCAAGTTGTCATGTCTTTTCA	80	83	This study
**FRK**	BQ510934 ^a^; Sotub03g007180 ^b^	FP: TGCCCTGACAGTGACAGGAA; RP: GCAGTGACCTCGGCAAGAGT; Probe: TCCCATCCCTTCCTACACAAGATGCA	84	112	This study
**HKS**	AF106068 ^a^; Sotub04g034770 ^b^; Sotub11g020550 ^b^	FP: GTCTTTTATGCATTGGATCTTGGT; RP: ACCATCTTTTCCMCCCAATT	72	89	This study
**GAD**	BQ514644 ^a^; Sotub11g007320 ^b^	FP: TGGAGTTGGAACAGTTGGATCTT; RP: GCTTTCATTTTGTTTTGCCATTT; Probe: AGCCATTATGCTTGCTGGATTGGCCTTT	83	92	This study
**SSADH**	TA26508_4113 ^d^;Sotub09g025220 ^b^	FP: GCAGATCTTGAAGTAGCTTTAAAAGGA; RP: TCGCGCATACACATGTTTGTC; Probe: CTCTGGCAACAAAGTTCCGTAACACC	76	94	This study
**PAL**	MICRO.2219.C3 ^c^; Sotub09g007470 ^b^	FP: CCTCCGTGGMACGVTCAC; RP: TGAGCAAACCAGCAATGTAGGA; Probe: CCTCRGGTGATCTTGTCCCTC	65	89	This study
**C4H**	TA25065_4113 ^d^; Sotub06g033350 ^b^	FP: TTGCAAATACCAAGAGCATGGA; RP: TCCCTTCTGTTG AGCATCAAGA	77	99	This study
**CWINV**	STMIM75TV ^c^; Sotub10g025820.1.1 ^b^	FP: CGCGGAGAGAATCACAATTGA; RP: TCTCCCAATGTTCTAGTGCAACTTT; Probe: CTAAATGCTTGGAGCATGGCTAATG	75	102	[52]
**EF-1**	MICRO.37.C4 ^c^; MICRO.37.C14 ^c^; Sotub06g010680.1.1 ^b^	FP: GGAAGCTGCTGAGATGAACAAGA; RP: CTCACGTTCAGCCTTAAGTTTGTC; Probe: TCATTCAAGTATGCCTGGGTGCT	72	91	[8]
**COX**	X83206 ^a^; Sotub04g015050 ^b^	FP: CGT CGC ATT CCA GAT TAT CCA; RP: CAA CTA CGG ATA TAT AAG AGC CAA AAC TG; Probe: TGC TTA CGC TGG ATG GAA TGC CCT	74	99	[53]
**PVYuni**	AJ390300 ^a^	FP: CATAGGAGAAACTGAGATGCCAACT; RP: TGGCGAGGTTCCATTTTCA; Probe: TGATGAATGGGCTTATGGTTTGGTGCA	73	100	[24]

BAM, β-amylase; FRK, fructokinase; HKS, hexokinase; GAD, glutamate decarboxylase; SSADH, succinic semialdehyde dehydrogenase; PAL, phenylalanine-ammonia lyase; C4H, cinnamate 4-hydroxylase; CWINV, cell-wall invertase; EF-1, elongation factor 1; COX, cytochrome oxidase; PVYuni, Potato virus Y universal amplicon. Sequences are available on

^a^ NCBI,

^b^ http://solcyc.solgenomics.net/,

^c^ POCI database;

^d^ ftp://occams.dfci.harvard.edu/pub/bio/tgi/data/Solanum_tuberosum/.

For the assay design, potato sequences from The Institute for Genomic Research (TIGR) transcript assembly database (ftp://occams.dfci.harvard.edu/pub/bio/tgi/data/Solanum_tuberosum/), the Potato Oligo Chip Initiative (POCI) database (http://pgrc-35.ipk-gatersleben.de/pls/htmldb_pgrc/f?p=194:1), and the Potato Genome Sequencing Consortium (PGSC) database (http://www.potatogenome.net/index.php/Main_Page) were aligned in the VectorNTI software (InforMax), and the regions of homology were determined. Primers were designed using the PrimerExpress software (Applied Biosystems) (see Table 1). The concentrations of primers and probes were optimised. If needed, dissociation curve analysis was carried out, and primer dimer formation was examined.

Universal Master Mix (TaqMan or SybrGreen, Applied Biosystems) together with primers and probes were used for the analysis of 2 μl cDNA. The final concentrations of all of the probes in the reaction mix was 150 nM, while the primer concentration was 300 nM, except for PVY-uni and reference amplicons COX and EF-1, where it was 250 nM and 900 nM for probe and primers, respectively. The probes for all of the newly designed amplicons were modified with the 5’-FAM reporter and with the 3'-Zen Iowa Black FQ quencher.

A 10-fold dilution series standard curve (10-times to 10000-times diluted) was prepared from a pool of all cDNA samples and tested with all amplicons. Ten-fold and 100-fold diluted cDNA was tested for each sample in two parallel reactions to check for the inhibition of amplification. All RT-qPCRs were carried out in 384-well reaction plates on a Lightcycler LC480 apparatus (Roche). For the amplifications, standard cycling conditions were used with an added melting curve stage for the SybrGreen reactions.

The data were first analysed using the Lightcycler 480 software (Roche). For quantification of the relative gene expression the standard curve method was used, as previously proposed by Pfaffl [54]. The efficiency of amplification of each amplicon was extrapolated from the slope of the standard curve. The data for the gene expression analysis were calculated as relative transcript copy numbers scaled to COX or the geometrical mean of both of the reference genes.

### Extraction of metabolites and GC-MS analysis

Plant material (50 mg) from the inoculated third leaf (from the ground) and a non-inoculated leaf were used separately for the extraction of the metabolites. Chilled (-20°C; 1 ml) methanol: chloroform: water (5:2:1; v/v/v) was added to the plant material that was homogenised in liquid nitrogen, and this was vortexed for 10 s and incubated on ice for 8 min. Samples were centrifuged at 14,000× *g* for 4 min at 4°C. The supernatant was transferred to a new tube, and 500 μl MilliQ water was added to create two separate phases. The samples were briefly vortexed and then centrifuged at 14,000× *g* for 2 min at 4°C. The aqueous upper phase (500 μl) was transferred to a new tube, dried in a speed-vac, and stored at -20°C until derivatisation. Five microlitres of ^13^C-sorbitol standard (0.1 g/l) was added to the dried samples, which were dried again in the speed-vac. The samples were resuspended in 20 μl 40% methoxyaminohydrochloride in pyridine (w/v). The mixture was incubated and agitated for 90 min at 30°C. After this incubation, 80 μl N-methyl-N-(trimethylsilyl)trifluoroacetamide (with added retention index marker of alkanes C10-C40, at 1.46 mg/l) was added to the samples. The samples were incubated at 37°C for 30 min, and then centrifuged at 14,000× *g* for 2 min at room temperature. Fifty microlitres of the supernatant was the transferred to the GC microvials.

The GC-MS analysis was carried out as described by [55]. For the GC-MS data analysis, the AMDIS, LCquan and Xcalibur software were used (Thermo Fisher Scientific Inc.). Chromatograms for each sample were first deconvoluted using AMDIS. Separate peaks were further identified in AMDIS and in Xcalibur. For peak identification, our in-house mass spectra library and the Golm Metabolite Database (http://gmd.mpimp-golm.mpg.de/) were used. Representative ions were selected for quantification. The peaks were quantified using the LCquan software, and the data were exported to MS Excel. To test the reliability of the selected ions for some of the metabolites, two ions were selected for quantification (e.g., serine 3TMS, ions *m/z* 204 and *m/z* 278). The same data were obtained regardless of the ions selected, and the quantity of one ion was used for further analysis. The identified metabolites were annotated with MapMan ontology (http://mapman.gabipd.org/web/guest/mapman), and new potato metabolites were added to MapMan.

### Data analysis

To determine the metabolite composition in different samples under the different treatments (i.e., mock, PVY^N^, PVY^NTN^) and harvested at three different times (i.e., 1, 3, 6 dpi), distance matrix analysis and hierarchical clustering (Pearson correlation, average linkage clustering) was carried out using MultiExperiment Viewer (http://www.tm4.org/mev.html), where the table of all of the determined peaks and set of identified metabolites only in each individual sample was used, respectively. In addition the PCA was carried out on the set of all determined peaks from 1 and 6 dpi in XLSTAT add-in for MS Excel (http://www.xlstat.com/en/).

For the relative levels of the metabolites, the means of four biological replicates were calculated. Ratios were determined for PVY^N^/mock, PVY^NTN^/mock and PVY^NTN^/PVY^N^ inoculated plants, and log2 transformed. The same procedure was used for determination of the relative transcript copy number. The mean transcript copy numbers of four biological replications were calculated, and the ratios between the differently treated samples were determined, and log2 transformed.

Two-way ANOVA was applied to determine which metabolites showed statistically significant changes in relation to time or treatment, and their combination. ANOVA was carried out in MultiExperiment Viewer (http://www.tm4.org/mev.html). For determining the statistically significant changes in metabolite concentrations and in transcript copy numbers, the data were also analysed using two-sided Student *t*-tests implemented in MS Excel. The cut-off *p*-value for statistical significance was set to <0.05.

### Data integration and visualisation

The MapMan potato mapping file was used for annotation of the transcripts with their associated metabolic pathways. Metabolites and their annotations were added to the mapping file. For visualisation of the data, a figure showing the selected metabolic pathways was prepared and integrated into the MapMan software.

## Supporting Information

S1 Fig Inoculated leaves of potato plants of cv. Igor at 7 dpi. No symptoms were observed on the mock-inoculated leaves (left), no symptoms or mild clorotic or necrotic ring spot lesions on the PVY^N^-inoculated leaves (middle), and more pronounced chlorotic and necrotic ring spot lesions on the PVY^NTN^-inoculated leaves (right).
(TIF)

S2 Fig GC-MS chromatogram of metabolites extracted from PVY^NTN^ inoculated potato leaf harvested at 6 days after inoculation. The chromatogram is shown in AMDIS software. The total ion chromatogram (TIC) represents all ions eluting in a scan of the potato sample (upper panel). Chromatogram of an ion *m/z* 347 is shown in red. The spectrum of a compound eluted at 27.0 minutes is shown on the middle panel, where raw spectrum (black ions) is overlaid with extracted spectrum after deconvolution (white ions). Hit from the library (black ions, citric acid) overlaid with extracted spectrum (white ions) is on the lower panel.
(TIF)

S3 Fig Principal component analysis plot according to the metabolite profiles of the 1 dpi and 6 dpi samples. Symbols: white, mock inoculated; grey, PVYN inoculated; black, PVYNTN inoculated; circles, 1 dpi; squares, 6 dpi. Clear separation can be seen for the first component (F1) for the samples collected at the two different times (elipse, 1 dpi; rectangles, 6 dpi), with separation of the three out of four mock-inoculated samples at 6 dpi from all of the other samples according to the second component (F2).
(DOC)

S1 Table Amount of metabolites detected at the different times after the inoculations in lower and upper potato leaves. Amount of individual metabolite, normalised to internal standard is shown.
(XLSX)

S2 Table Log2 ratios of the metabolites detected in the potato leaves at the different times (i.e., 1, 3, 6 dpi) after the PVY inoculations. The ratios were calculated between the PVYN-inoculated and mock-inoculated leaves (N:mock), the PVYNTN-inoculated and mock-inoculated leaves (NTN:mock), and between the virus isolates used for the PVY-inoculated plants (NTN:N). S, significance determined by student t-tests. Significance determined by two factor ANOVA is also given, where the time and treatment (treat) were given as factors and the significance of the interactions (inter) between these factors is given as well. *, p <0.05; **, p <0.01; ***, p <0.001.
(XLSX)

S3 Table Log2 ratios of the gene expression in the potato leaves at the different times (i.e., 1, 3, 6 dpi) after the PVY inoculations. The ratios were calculated between the PVYN-inoculated and mock-inoculated leaves (N:mock), the PVYNTN-inoculated and mock-inoculated leaves (NTN:mock), and between the virus isolates used for the PVY-inoculated plants (NTN:N). The corresponding metabolic pathways are assigned. CHO, carbohydrates; S, significance determined by student t-tests. *, p <0.05; **, p <0.01.
(XLS)

S4 Table Details on RT-qPCR reactions. (XLSX)
