# Supplementary figures and images for: Primary Metabolism, Phenylpropanoids and Antioxidant Pathways Are Regulated in Potato as a Response to Potato virus Y Infection
Source: PLoS One. 2016 Jan 4;11(1):e0146135. doi: 10.1371/journal.pone.0146135 (PMC4738437; doi:10.1371/journal.pone.0146135)

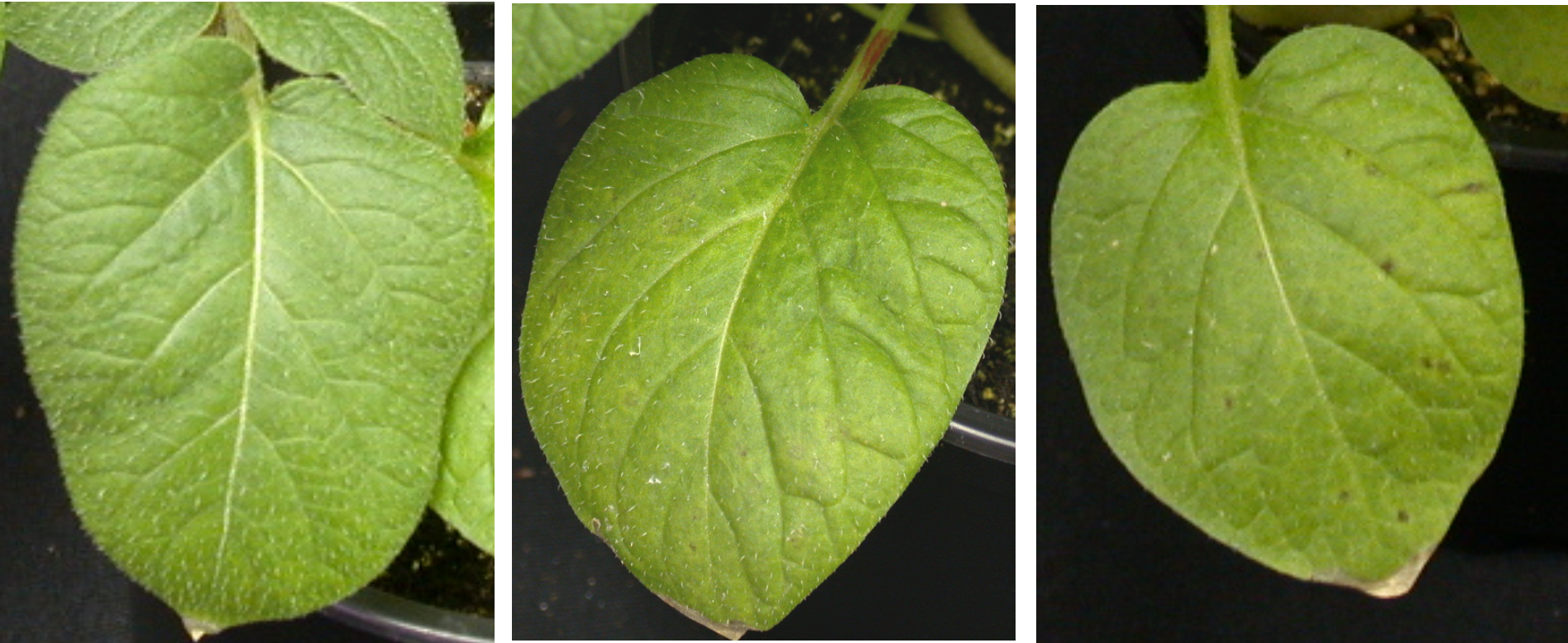

Supplement: S1 Fig — No symptoms were observed on the mock-inoculated leaves (left), no symptoms or mild clorotic or necrotic ring spot lesions on the PVYN-inoculated leaves (middle), and more pronounced chlorotic and necrotic ring spot lesions on the PVYNTN-inoculated leaves (right). (TIF) [file pone.0146135.s001.tif]

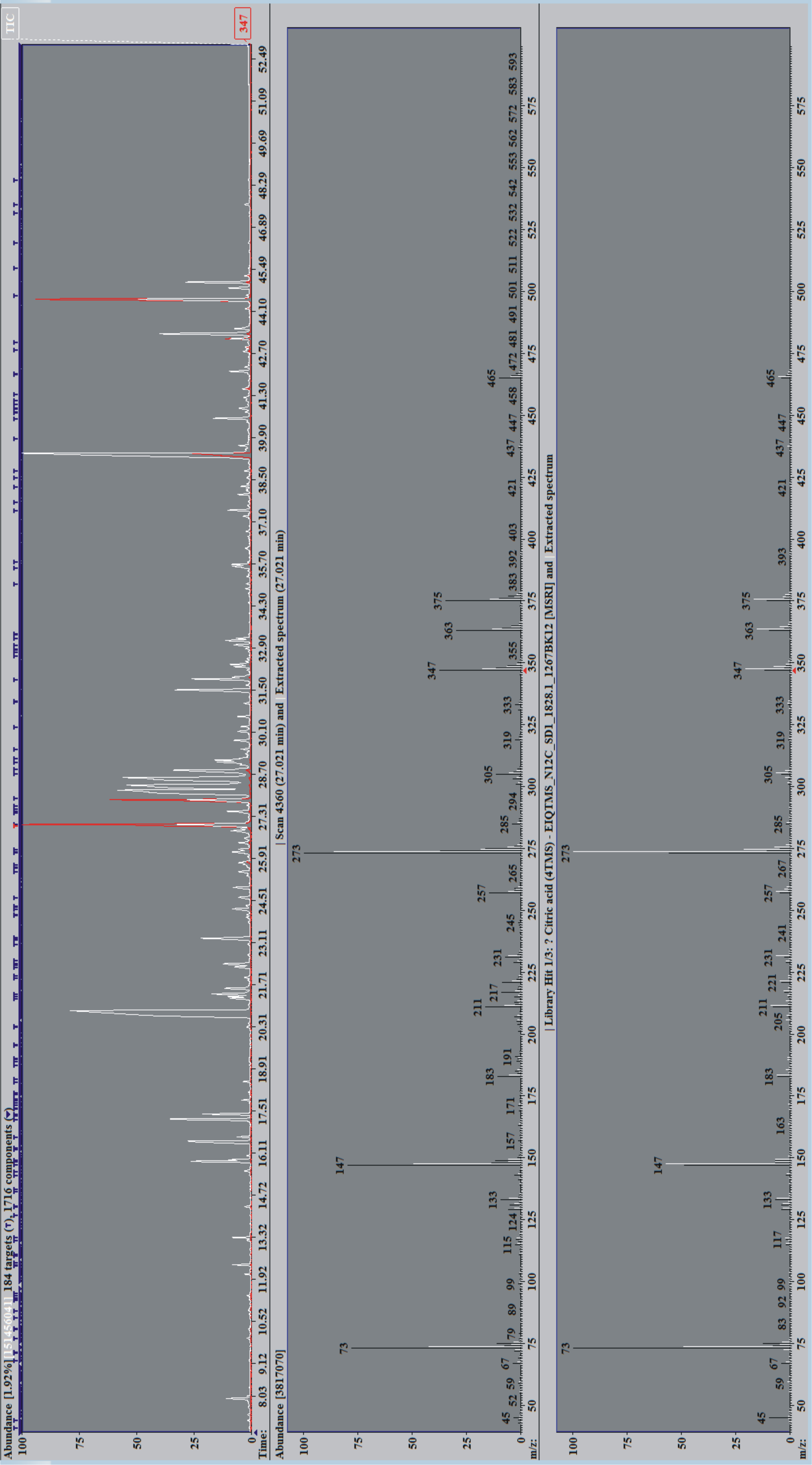

Supplement: S2 Fig — The chromatogram is shown in AMDIS software. The total ion chromatogram (TIC) represents all ions eluting in a scan of the potato sample (upper panel). Chromatogram of an ion m/z 347 is shown in red. The spectrum of a compound eluted at 27.0 minutes is shown on the middle panel, where raw spectrum (black ions) is overlaid with extracted spectrum after deconvolution (white ions). Hit from the library (black ions, citric acid) overlaid with extracted spectrum (white ions) is on the lower panel. (TIF) [file pone.0146135.s002.tif]

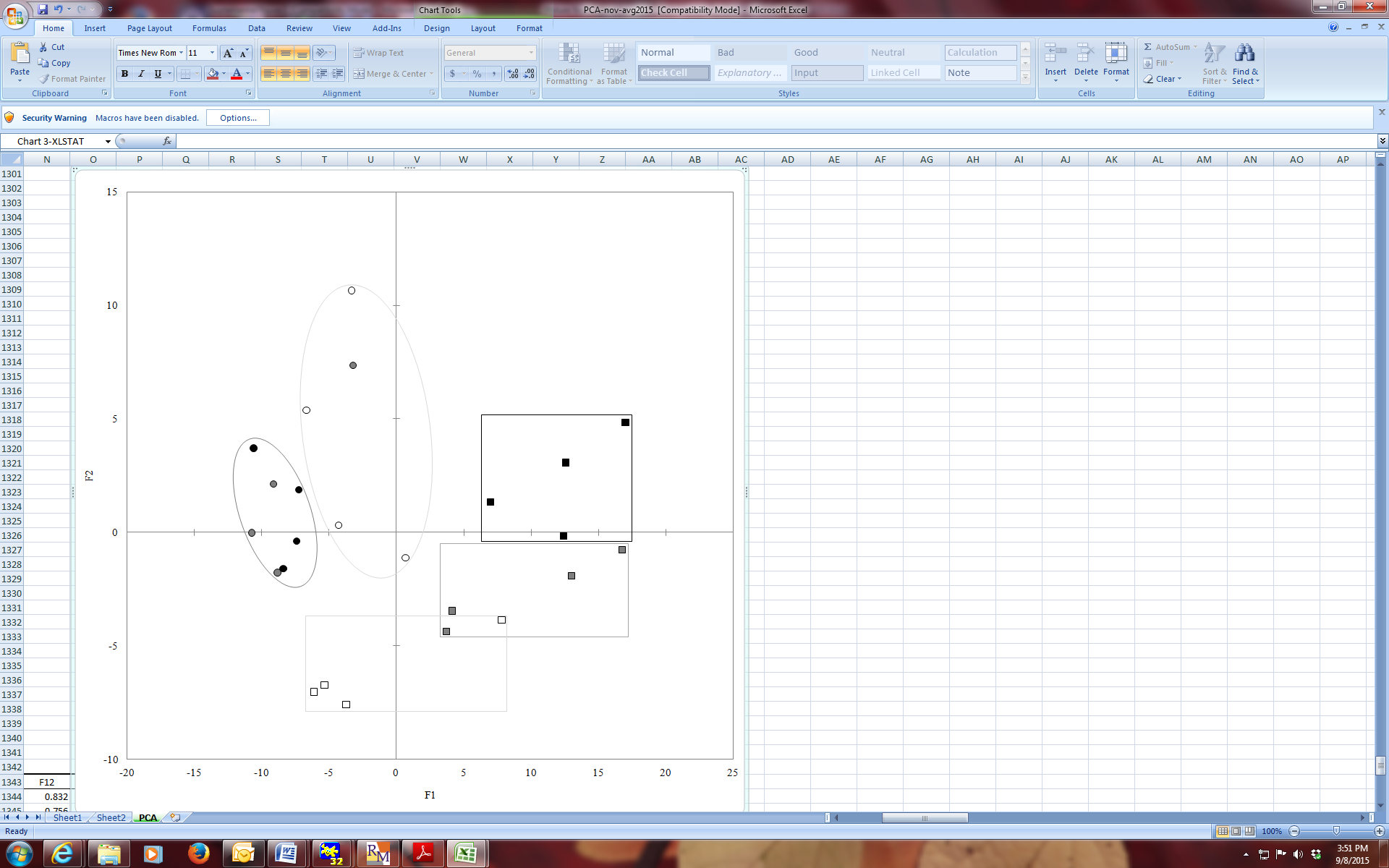

Supplement: S3 Fig — Symbols: white, mock inoculated; grey, PVYN inoculated; black, PVYNTN inoculated; circles, 1 dpi; squares, 6 dpi. Clear separation can be seen for the first component (F1) for the samples collected at the two different times (elipse, 1 dpi; rectangles, 6 dpi), with separation of the three out of four mock-inoculated samples at 6 dpi from all of the other samples according to the second component (F2). (DOC) [file pone.0146135.s003.doc]
